# Genome-Resolved Proteomic Stable Isotope Probing of Soil Microbial Communities Using ^13^CO_2_ and ^13^C-Methanol

**DOI:** 10.3389/fmicb.2019.02706

**Published:** 2019-12-06

**Authors:** Zhou Li, Qiuming Yao, Xuan Guo, Alexander Crits-Christoph, Melanie A. Mayes, William Judson Hervey IV, Sarah L. Lebeis, Jillian F. Banfield, Gregory B. Hurst, Robert L. Hettich, Chongle Pan

**Affiliations:** ^1^Computer Science and Mathematics Division, Oak Ridge National Laboratory, Oak Ridge, TN, United States; ^2^Graduate School of Genome Science and Technology, The University of Tennessee, Knoxville, Knoxville, TN, United States; ^3^Chemical Sciences Division, Oak Ridge National Laboratory, Oak Ridge, TN, United States; ^4^Department of Plant and Microbial Biology, University of California, Berkeley, Berkeley, CA, United States; ^5^Environmental Sciences Division, Oak Ridge National Laboratory, Oak Ridge, TN, United States; ^6^Naval Research Laboratory, Center for Biomolecular Science and Engineering, Washington, DC, United States; ^7^Department of Microbiology, The University of Tennessee, Knoxville, Knoxville, TN, United States; ^8^Department of Earth and Planetary Science, University of California, Berkeley, Berkeley, CA, United States; ^9^Department of Environmental Science, Policy, and Management, University of California, Berkeley, Berkeley, CA, United States; ^10^School of Computer Science and Department of Microbiology and Plant Biology, University of Oklahoma, Norman, OK, United States

**Keywords:** stable isotope probing, metagenomic analyses, metaproteomic analysis, microbial ecology, rhizosphere

## Abstract

Stable isotope probing (SIP) enables tracking the nutrient flows from isotopically labeled substrates to specific microorganisms in microbial communities. In proteomic SIP, labeled proteins synthesized by the microbial consumers of labeled substrates are identified with a shotgun proteomics approach. Here, proteomic SIP was combined with targeted metagenomic binning to reconstruct metagenome-assembled genomes (MAGs) of the microorganisms producing labeled proteins. This approach was used to track carbon flows from ^13^CO_2_ to the rhizosphere communities of *Zea mays*, *Triticum aestivum*, and *Arabidopsis thaliana*. Rhizosphere microorganisms that assimilated plant-derived ^13^C were capable of metabolic and signaling interactions with their plant hosts, as shown by their MAGs containing genes for phytohormone modulation, quorum sensing, and transport and metabolism of nutrients typical of those found in root exudates. XoxF-type methanol dehydrogenases were among the most abundant proteins identified in the rhizosphere metaproteomes. ^13^C-methanol proteomic SIP was used to test the hypothesis that XoxF was used to metabolize and assimilate methanol in the rhizosphere. We detected 7 ^13^C-labeled XoxF proteins and identified methylotrophic pathways in the MAGs of 8 ^13^C-labeled microorganisms, which supported the hypothesis. These two studies demonstrated the capability of proteomic SIP for functional characterization of active microorganisms in complex microbial communities.

## Introduction

Plants release a substantial fraction of their photosynthetically fixed carbon from roots into rhizosphere as carbohydrates, amino acids, organic acids, and other compounds (Zhalnina et al., 2018). These carbon substrates can be consumed by microorganisms in the rhizosphere communities. In return, rhizosphere communities help plants extract nutrients from soil (Bulgarelli et al., 2013), modulate plant growth with phytohormones (Costacurta and Vanderleyden, 1995), and defend plants against soil-borne pathogens (Mendes et al., 2011). Studies of rhizosphere communities have shed light on the cycling of carbon and nutrients between plants and soil in many natural terrestrial ecosystems (Jones et al., 2009). However, microbe-plant interactions in the rhizosphere are still poorly understood owing to the immense complexity of rhizosphere communities and the difficulty of comprehensively cultivating rhizosphere microorganisms from diverse taxa (Bulgarelli et al., 2012; Lundberg et al., 2012; Lebeis et al., 2015).

Cultivation-independent stable isotope probing (SIP) has been used to track carbon flows from plants to specific members of rhizosphere communities (el Zahar Haichar et al., 2016). Growing under a ^13^CO_2_ atmosphere, plants fix ^13^C and release ^13^C-labeled compounds into the rhizosphere. Microorganisms that directly assimilate these ^13^C-labeled compounds should produce ^13^C-labeled biomass, including DNA, RNA, and proteins. In previous studies (Lu and Conrad, 2005; Vandenkoornhuyse et al., 2007; el Zahar Haichar et al., 2008; Drigo et al., 2010), ^13^C-labeled DNAs and RNAs were isolated by density gradient ultracentrifugation and then amplicon-sequenced to identify the microbial taxa that assimilated ^13^C in rhizosphere communities. For example, a DNA SIP study showed that bacteria in the order *Sphingobacteriales* and the genus *Myxococcus* assimilated plant-derived carbon in the rhizosphere of wheat, maize, rapeseed, and medicago (el Zahar Haichar et al., 2008). RNA SIP was used to uncover the carbon transfer from rice to a rhizosphere methanogen belonging to Rice Cluster I Archaea (Lu and Conrad, 2005) and from white clover to arbuscular mycorrhizal fungi (Vandenkoornhuyse et al., 2007). Recently, a genome-resolved metagenomic approach was implemented in a DNA SIP study which recovered a complete genome for *Saccharibacteria* (TM7) from the rhizosphere of wild oats (Starr et al., 2018).

In comparison with DNA SIP and RNA SIP, proteomic SIP has the advantages of accurate atom% estimation and a low detection limit for the labeling levels (as low as 2% for ^13^C labeling) (Pan et al., 2011; Justice et al., 2014; Bryson et al., 2016, 2017; Marlow et al., 2016), because it is based on precise measurement of the mass shifts of labeled peptides. However, proteomic SIP provides less direct information than DNA SIP or RNA SIP for taxonomy identification of the labeled microorganisms. Binning of metagenome-assembled genomes (MAGs) has been shown in previous studies to be a powerful cultivation-independent method to recover novel genomes from complex metagenomics (Crits-Christoph et al., 2018; Delmont et al., 2018). Genome-resolved metagenomics and proteomics have been combined to survey the protein expression profiles of microbial communities (Ram et al., 2005). Protein-based SIP was used with a binned metagenome for characterization of ^13^C-acetate turnover in anaerobic digestion communities (Mosbaek et al., 2016). In this study, proteomic SIP was coupled with targeted metagenomic binning to identify ^13^C-labeled proteins and reconstruct MAGs of ^13^C-labeled microorganisms in a ^13^CO_2_ rhizosphere SIP experiment. Proteomic SIP and metagenomics also provided a cultivation-independent method to validating the substrates of enzymes used by microbial communities for carbon uptake. This method was used in a ^13^C-methanol SIP experiment to support methanol as a putative substrate of the abundant XoxF-type methanol dehydrogenase in the rhizosphere.

## Results

### ^13^CO_2_ Stable Isotope Probing, Metagenomic Sequencing, and Metaproteomics Analyses of Rhizosphere Communities

Three model plants, including *Z. mays*, *T. aestivum*, and *A. thaliana*, were cultivated in pots filled with the same initial soil collected from the field (4 pots for each plant species for a total of 12 pots) (Supplementary Figure 1). After 29 days of growth in the normal atmosphere, the plants were moved to grow in a labeling chamber with a ^13^CO_2_ atmosphere (99% atom% ^13^C). Duplicate pots from each plant species were harvested after 3 days of ^13^CO_2_ labeling to generate timepoint-1 (T1) rhizosphere samples. The remaining duplicate pots from each plant species were harvested after 8 days of ^13^CO_2_ labeling to generate timepoint-2 (T2) rhizosphere samples. The 12 rhizosphere soil samples (3 plant species X 2 timepoints X 2 replicates) and 2 initial soil samples as the controls were measured with metagenomics and metaproteomics (Butterfield et al., 2016; Yao et al., 2018).

Because the 14 samples originated from the same initial soil, the 203 Gbp of metagenomic sequencing data (a total of 1.4 billion 2 × 150-bp reads) from all samples were merged and co-assembled into a composite metagenome (Supplementary Table 1). The composite metagenome contained 6.5 Gbp of scaffolds longer than 1 kbp with an N50 score of 2,331 bp. It encoded a total of 8.4 million predicted protein-coding genes. The metaproteomics measurements identified an average of 5,287 unlabeled microbial proteins or protein groups per soil sample with protein false discovery rates (FDRs) all less than 2% (Supplementary Table 1).

Proteomic SIP searches identified 19 ^13^C-labeled microbial proteins or protein groups with >10% ^13^C enrichment and no decoy ^13^C-labeled protein (Supplementary Table 2), which indicated a low FDR in the identification of ^13^C-labeled proteins. Furthermore, no ^13^C-labeled protein was identified from the 2 initial soil samples, which also supported the low FDR of the ^13^C-labeled protein identification in this study. This was consistent with the low FDR of proteomic SIP searches in our previous studies (Pan et al., 2011; Justice et al., 2014; Bryson et al., 2016, 2017; Marlow et al., 2016). The average ^13^C atom% of ^13^C-labeled proteins from the T2 rhizosphere samples was higher than that from the T1 rhizosphere samples (*p* value = 0.00262, Mann–Whitney *U* test). Most of the identified ^13^C-labeled proteins were abundant housekeeping proteins, such as ribosomal proteins, chaperones, and glycolysis enzymes.

### Reconstruction of MAGs for the Unlabeled and ^13^C-Labeled Rhizosphere Microorganisms

**FIGURE 1:**
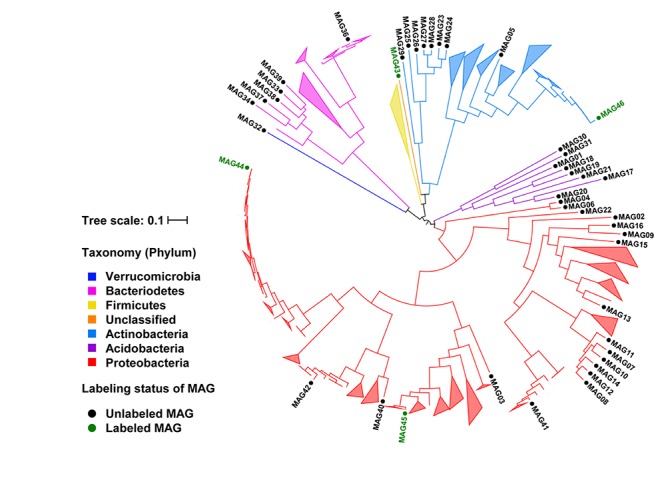
Phylogenomic tree of MAGs in the phylogenomics context of existing root-associated bacterial isolate genomes. Branches labeled with MAG IDs represent the 41 unlabeled and 4 labeled bacterial MAGs recovered from this study. The MAG35 from the Archaea was not shown on the tree. The remaining branches and all collapsed clades represent the 523 existing isolate genomes from Levy et al. (2018). Branches are color-coded based on the phylum-level taxonomy. MAGs for the ^13^C-labeled microorganisms in ^13^CO_2_ rhizosphere proteomic SIP are shown in green.

The abundance series of scaffolds in the composite metagenome assembly across the 14 soil samples were used to bin scaffolds into MAGs. The untargeted binning produced 41 medium-quality bacterial MAGs and 1 medium-quality archaeal MAG. All these MAGs had more than 70% genome completeness and less than 10% genome contamination (Supplementary Table 3). The 41 bacterial MAGs were analyzed in the phylogenomics context of 523 isolate genomes of root-associated bacteria from a previous study (Levy et al., 2018) (Figure 1). The 523 existing isolate genomes all belonged to four major bacterial phyla, including *Proteobacteria*, *Actinobacteria*, *Bacteroidetes*, and *Firmicutes*. Consistently, 32 of the 41 bacterial MAGs belonged to *Proteobacteria* (*n* = 18), *Actinobacteria* (*n* = 8), and *Bacteroidetes* (*n* = 6). Most of the MAGs formed distinct clades separate from the existing isolate genomes, including a clade of 7 MAGs in the *Actinobacteria* phylum, a clade of 5 MAGs in the *Sphingobacteriales* order of the *Bacteroidetes* phylum, and a clade of 6 MAGs in the *Sphingomonadales* order of the *Proteobacteria* phylum. Furthermore, 10 MAGs were recovered from phyla under-represented by the existing root-associated isolate genomes, including 8 bacterial MAGs from the *Acidobacteria* phylum, 1 bacterial MAG from the *Verrucomicrobia* phylum, and 1 archaeal MAG from the Thaumarchaeota phylum. Thus, the MAGs from our untargeted binning expanded the phylogenomic coverage and genomic diversity of root-associated bacteria and archaea.

Metagenome-assembled genomes of the rhizosphere microorganisms that produced ^13^C-labeled proteins were recovered using a targeted binning approach. The scaffolds containing the genes for ^13^C-labeled unique proteins were used as the binning targets to cluster other scaffolds around. Each obtained MAG was required to have a minimum of two independent identifications of ^13^C-labeled unique proteins in order to increase the confidence on the ^13^C-labeling status of the microorganisms. The contamination levels of the MAGs were controlled below 5%. Out of the 28 ^13^C-labeled protein identifications, 14 were binned into four MAGs, including the medium-quality MAG45 with genome completeness at 54% and three low-quality MAGs (MAG43, MAG44, and MAG46) with genome completeness between 21% and 42% (Table 1). The ^13^C atom% enrichment of the binned protein identifications ranged between 15% and 46%. MAG44 belonged to the *Pseudomonas* genus, MAG45 to the Oxalobacteraceae family, MAG46 to the *Arthrobacter* genus, and MAG43 to an unclassified taxon related to the Chloroflexi-related group (CHLX; Anantharaman et al., 2016). MAG44, MAG45, and MAG46 were closely related to other root-associated bacterial isolates (Figure 1).

**TABLE 1:** ^13^C-labeled proteins and microorganisms from ^13^CO_2_ rhizosphere proteomic SIP.

**Labeled protein (Protein ID)**	**Plant host**	**Time point**	**^13^C atom%**	**MAG ID**	**Completeness**	**Contamination**	**Taxonomy**	**Genome size (Mbp)**	**# of genes**
Glyceraldehyde-3-phosphate dehydrogenase (scaff_0000095019_7)	*Z*. *mays*	T1	24%	MAG43	21%	3.6%	Unclassified	0.7	692
	*Z. mays*	T2	15%						
	*T. aestivum*	T2	30%						

Cold shock protein CapA (scaff_0000016092_2)	*Z. mays*	T1	18%	MAG44	42%	1.8%	Pseudomonas	5.5	5207
Chaperone DnaK (scaff_0000160764_4)	*Z. mays*	T1	16%						

Protein with unknown function (scaff_0000030713_6)	*Z. mays*	T2	18%	MAG45	54%	0.3%	Oxalobacteraceae	2.9	2853
Colicin I receptor (scaff_0000049197_5)	*Z. mays*	T2	17%						

Enolase (scaff_0000017170_6)	*T. aestivum*	T2	20%	MAG46	29%	0%	Arthrobacter	2.3	2252
	*T. aestivum*	T2	28%						
Dihydrolipoyl dehydrogenase (scaff_0000125141_2)	*T. aestivum*	T1	16%						
	*T. aestivum*	T2	26%						
	*T. aestivum*	T2	46%						
Protein with unknown function (scaff_0000159039_6)	*T. aestivum*	T2	34%						
Chaperonin GroEL (scaff_0000063839_3)	*Z. mays*	T1	29%						

### Genetic Potential and Expressed Functions in the ^13^C-Labeled Rhizosphere Microorganisms

**FIGURE 2:**
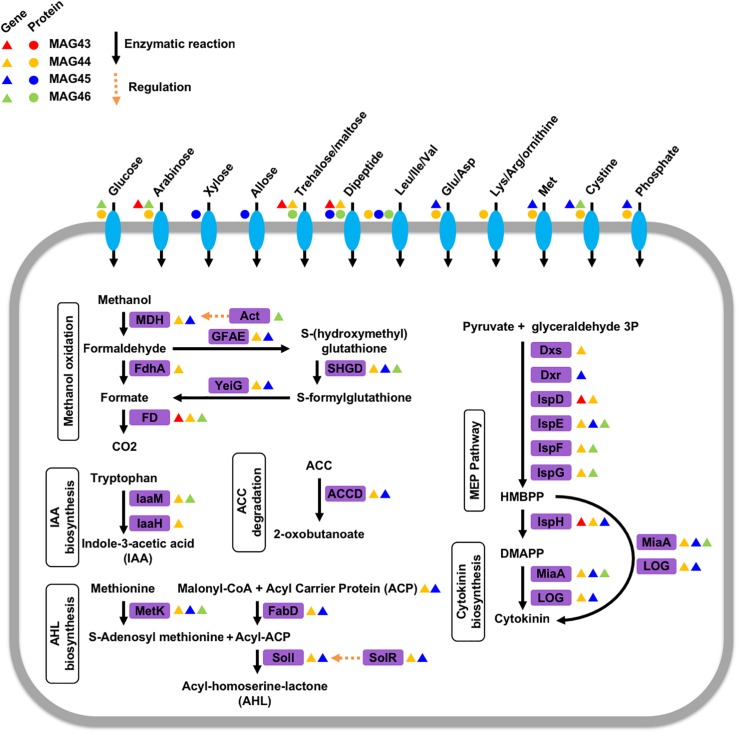
Phytohormone modulation, nutrient transportation, quorum sensing, and methanol metabolism in the four ^13^C-labeled microorganisms. Enzymes in purple boxes are labeled with triangles for encoded genes and circles for expressed proteins in these microorganisms. The triangles and circles are color-coded for each microorganism. MDH: NAD-dependent methanol dehydrogenase, Act: methanol dehydrogenase activator, FdhA: glutathione-independent formaldehyde dehydrogenase, FD: formate dehydrogenase, GFAE: glutathione-dependent formaldehyde-activating enzyme, SHGD: S-(hydroxymethyl)glutathione dehydrogenase, YeiG: S-formylglutathione hydrolase, laaM: tryptophan 2-monooxygenase, laaH: indole acetamide hydrolase, MetK: methionine adenosyltransferase, ACCD: 1-aminocyclopropane-1-carboxylate deaminase, FabD: malonyl CoA-acyl carrier protein transacylase, Soil: acyl-homoserine-lactone synthase, SoIR: transcriptional activator/quorum-sensing receptor, Dxs:1-deoxy-D-xylulose 5-phosphate synthase, Dxr: 2-C-methyl-D-erythritol 4-phosphate synthase, IspD: 2-C-methyl-D-erythritol 4-phosphate cytidylyltransferase, IspE: 4-diphosphocytidyl-2-C-methyl-D-erythritol kinase, IspF: 2-C-methyl-D-erythritol 2,4-cyclodiphosphate synthase, IspG: 1-hydroxy-2-methyl-2-(E)-butenyl 4-diphosphate synthase, IspH: 4-hydroxy-3-methylbut-2-enyl diphosphate reductase, MiaA: tRNA isopentenyltransferase, LOG: cytokinin-specific phosphoribohydrolase ‘Lonely guy’, HMBPP: (E)-4-Hydroxy-3-methyl-but-2-enyl pyrophosphate, DMAPP: dimethylallyl pyrophosphate.

The genetic potential and expressed functions of the four ^13^C-labeled rhizosphere microorganisms were inferred based on their genes contained in the MAGs and their unlabeled proteins identified by the regular label-free proteomics searches. These microorganisms encoded genes and had the capability to produce proteins for a variety of transporters of sugars and amino acids (Figure 2). We identified proteins for transporters of glucose and arabinose in MAG44, xylose and allose transporters in MAG45, and a trehalose/maltose transporter in MAG46. We also identified proteins for transporters of dipeptides and branched-chain amino acids (Leu/Ille/Val) in MAG45 and MAG46 and transporters of a variety of amino acids in MAG44. Identification of these transporter proteins in the ^13^C-labeled microorganisms suggested their extraction of sugars and amino acids from the rhizosphere soils. These MAGs also encoded genes for methanol oxidation through formaldehyde and formate (Chistoserdova et al., 2009) (Figure 2). The sugars, amino acids, and/or methanol may carry plant-derived ^13^C to these microorganisms.

Rhizosphere microorganisms can modulate the physiology of their plant hosts by producing phytohormones, such as cytokinin and auxin. MAG44 and MAG45 encoded the genes for tRNA: isopentenyltransferase (MiaA) and cytokinin-specific phosphoribohydrolase ‘Lonely guy’ (LOG) in the cytokinin synthesis pathway (Frébort et al., 2011) (Figure 2). MAG46 also encoded the gene for MiaA. This was consistent with a previous rhizosphere study (Starr et al., 2018). The substrates for cytokinin synthesis can be produced through the 2-C-methyl-D-erythritol 4-phosphate (MEP) pathway (Frébort et al., 2011). Out of the seven genes in the MEP pathway, 6 were found in MAG44, 3 in MAG45 and MAG46, and 2 in MAG43. The absence of the other genes in this pathway may be attributed to the low completeness of the MAGs.

Indole-3-acetic acid (IAA) is a major type of auxin and can be produced from tryptophan using a tryptophan monooxygenase (IaaM) and an indole-3-acetamide hydrolase (IaaH) (Patten and Glick, 1996). A gene encoding IaaM was found in MAG44 and MAG46 (Figure 2). In MAG44, the *iaaM* gene was adjacent to a carbon-nitrogen hydrolyase gene (E.C. 3.5.-.-), which may carry out the function of IaaH (E.C. 3.5.1.-). MAG44 also encoded genes for a two-component system GacS/GacA and a sigma factor RpoS, which can regulate the transcription of IaaM and IaaH (Bulgarelli et al., 2013).

Plants can synthesize ethylene to regulate their growth (Morgan and Drew, 1997). 1-aminocyclopropane-1-carboxylate (ACC) is a precursor for ethylene synthesis (Glick et al., 2007). ACC can be exuded by plant roots and degraded into α-ketobutyrate and ammonia by the ACC deaminase (ACCD) in rhizosphere microorganisms (Bulgarelli et al., 2013). MAG44 and MAG45 both possessed a gene for the ACCD (Figure 2). The degradation of ACC by MAG44 and MAG45 may lower the ethylene level inside their plant hosts to attenuate the ethylene-dependent growth inhibition (Bulgarelli et al., 2013).

Rhizosphere microorganisms can use acyl-homoserine-lactone (AHL) as a quorum-sensing autoinducer (DeAngelis et al., 2008). Both MAG44 and MAG45 contained the gene for an AHL synthase, SolI. The *solI* gene in both MAGs was adjacent to a *solR* gene which encodes a AHL receptor that can activate the transcription of *solI* (Lerat and Moran, 2004). Both MAG44 and MAG45 also encoded the genes for S-adenosylmethionine synthetase (MetK), an acyl-carrier protein (ACP), and malonyl CoA-acyl carrier protein transacylase (FabD) for AHL synthesis (Figure 2). The presence of the AHL synthesis genes and the AHL receptor gene in MAG44 and MAG45 indicated the quorum sensing capability in these two microorganisms.

### Protein Expression Profiles of the Rhizosphere Communities for Carbon Metabolism

Besides the ^13^C-labeled proteins, metaproteomics identified between 4,165 and 7,258 unlabeled proteins or protein groups from the 12 rhizosphere communities, which were used to characterize the expressed molecular functions at the community level. Protein identifications were aggregated into biological functions represented by gene ontology (GO) terms and EC numbers (Yao et al., 2018). Out of 5,200 GO terms and 2,569 EC numbers encoded in the metagenome, 1,352 GO terms and 509 EC numbers had protein expression detected and quantified by metaproteomics (Supplementary Figure 2).

**FIGURE 3:**
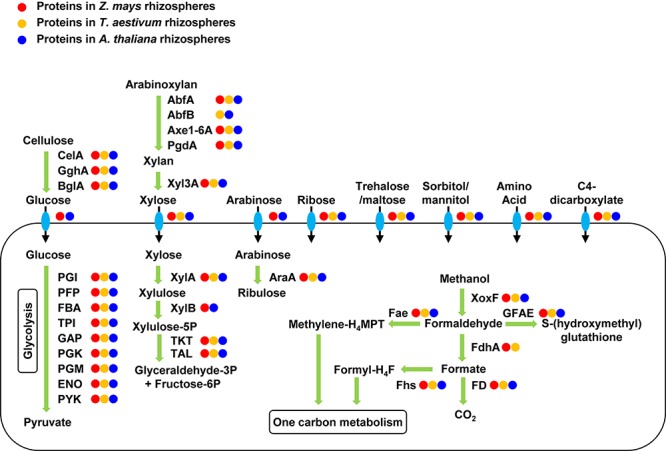
Expressed proteins for carbon uptake and metabolism in the rhizosphere communities. Enzymes are labeled with circles in three different colors for their protein expression in the rhizospheres of the three different plants. The rhizosphere communities expressed proteins for the transportation and metabolism of many complex carbohydrates and methanol. CelA, endo-1,4-beta-D-glucanase; GghA, exo-1,4-beta-glucosidase; BgIA, beta-glucosidase; PGI, phosphoglucose isomerase; PFP, pyrophosphate-dependent fructose-6P 1-phosphotransferase; FBA, fructose-bisphosphate aldolase; TPI, triose-phosphate isomerase; GAP, glyceraldehyde-3P dehydrogenase; PGK, phosphoglycerate kinase; PGM, phosphoglycerate mutase; ENO, enolase; PYK, pyruvate kinase; AbfA, alpha-arabinosidase; AbfB, alpha-arabinosidase; Axe1-6A, feruloyl esterase; PgdA, acetylxylan esterase; Xyl3A, xylan 1,4-beta-xylosidase; XylA, xylose isomerase; XylB, xylulose kinase; TKT, transketolase; TAL, transaldolase; AraA, arabinose isomerase; XoxF, methanol dehydrogenase; FdhA, glutathione-independent formaldehyde dehydrogenase; FD, formate dehydrogenase; GFAE, glutathione-dependent formaldehyde-activating enzyme, Fae, 5,6,7,8-tetrahydromethanopterin hydro-lyase; Fhs, formate-tetrahydrofolate ligase.

The rhizosphere communities expressed proteins for degrading cellulose, xylan, and arabinoxylan (Figure 3). Cellulose can be deconstructed to glucose by the identified endo-1,4-beta-D-glucanase (CelA), exo-1,4-beta-glucosidase (GghA), and beta-glucosidase (BglA) in the rhizosphere metaproteomes. Glucose can be transported into cells by the expressed glucose porin and glucose ABC transporter and be degraded through an active glycolysis pathway. Arabinoxylan can be debranched to xylan by the identified alpha-arabinosidase (AbfA and AbfB), feruloyl esterase (Axe1-6A), and acetylxylan esterase (PdfA). Xylan can be depolymerized to xylose by the expressed xylan 1,4-beta-xylosidase (Xyl3A). Xylose can be transported into cell by the expressed transporters, XylT and XylF, and be degraded by the identified xylose isomerase (XylA), xylulose kinase (XylB), and transketolase and transaldolase in the pentose phosphate pathway. Metaproteomics also identified transporters of other compounds, including arabinose, ribose, trehalose/maltose, sorbitol/mannitol, C4-dicarboxylate, and many amino acids. These compounds extracted from the rhizosphere soil may be important carbon substrates for the rhizosphere communities.

Metaproteomics identified 68 XoxF-type methanol dehydrogenases out of a total of 567 XoxF genes encoded in the metagenomes. The aggregate abundance of the identified XoxF proteins accounted for an average of 11% of the total abundance of all identified proteins in the metaproteomes. The phylogenetics of the 68 identified XoxF proteins was analyzed using 146 reference XoxF sequences (Taubert et al., 2015), which categorized the identified XoxF proteins into 4 groups, namely XoxF1, XoxF2, XoxF3, and XoxF5 (Supplementary Figure 3). Metaproteomics also identified glutathione-independent formaldehyde dehydrogenase (FdhA) and formate dehydrogenase (FD) for oxidation of formaldehyde and formate, respectively, in the methanol oxidation pathway to CO_2_ (Figure 3). Formaldehyde and formate can also be converted to methylene-tetrahydromethanopterin (methylene-H_4_MPT) and formyl-tetrahydrofolate (formyl-H_4_F) for the one-carbon metabolism by the identified H_4_MPT hydro-lyase (Fae) and formate-H_4_F ligase (Fhs), respectively (Figure 3).

### Testing of the Methylotrophic Metabolism With ^13^C-Methanol Proteomic SIP

Because of the existence of diverse XoxF proteins with high abundance across the rhizosphere and initial soils, we hypothesized that methanol was an important carbon source for many of these soil microorganisms via XoxF-based methylotrophic pathways. We tested this hypothesis using a ^13^C-methanol proteomic SIP experiment. A fresh sample of the initial soil was collected and ∼6.1 micromoles of ^13^C-methanol per gram of soil was added daily into six replicates of the fresh initial soil. A triplicate of samples was collected after 3 days of ^13^C-methanol addition and incubation and another triplicate was collected after 8 days (Supplementary Figure 4). Proteomic SIP searches detected 22 ^13^C-labeled microbial proteins or protein groups from 48 protein identifications in the soil samples amended with ^13^C-methanol (Supplementary Table 4). No decoy ^13^C-labeled protein was identified in any sample. This indicated that soil microorganisms incorporated carbon from methanol into their proteomes. Eighteen of these 48 ^13^C-labeled protein identifications were XoxF proteins, suggesting that the addition of methanol in the soils stimulated the biosynthesis of new XoxF proteins in the microorganisms that assimilated methanol. In addition, we identified a ^13^C-labeled membrane-bound aldehyde dehydrogenase which can oxidize formaldehyde to formate and a ^13^C-labeled H_4_MPT hydro-lyase for converting formaldehyde to methylene-H_4_MPT following the methanol oxidation.

**FIGURE 4:**
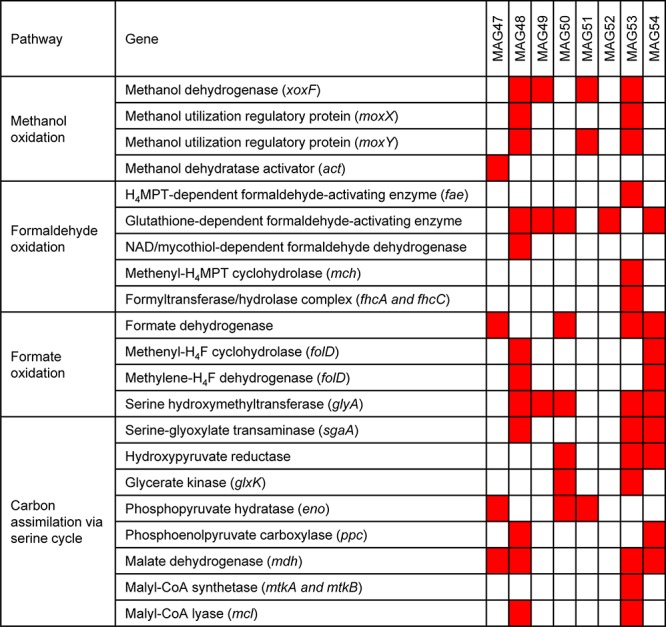
Methylotrophic metabolism in the ^13^C-labeled microorganisms from the ^13^C-methanol proteomic SIP experiment. ^13^C-methanol can be metabolized and assimilated by the eight ^13^C-labeled microorganisms through methanol oxidation, formaldehyde oxidation, formate oxidation, and the serine cycle. The red squares mark the presence of the genes involved in these pathways in the eight MAGs.

Scaffolds containing these ^13^C-labeled proteins were used as targets for metagenomic binning, which generated one medium-quality MAGs and seven low-quality MAGs with genome contamination below 5% (Table 2). Each of these MAGs had ^13^C-labeled protein identifications from at least two replicates in a ^13^C-methanol SIP incubation triplicate. Four of the eight MAGs contained a *xoxF* gene (Figure 4). Among these four MAGs, three produced ^13^C-labeled XoxF proteins and the remaining one expressed an unlabeled XoxF protein. All 8 ^13^C-labeled MAGs encoded many genes involved in the oxidation of methanol to formaldehyde and format and the carbon assimilation via the serine cycle (Figure 4). For example, the MAG53 encoded genes for methanol oxidation (*xoxF*), a two-component system involved in regulating expression of methanol dehydrogenase (*moxX* and *moxY*) (Harms et al., 1993), H_4_MPT-dependent formaldehyde oxidation, formate oxidation, and serine cycle for carbon assimilation (serine-glyoxylate transaminase, hydroxypyruvate reductase, glycerate 3-kinase, malate dehydrogenase, malyl-CoA synthetase, and malyl-CoA lyase) (Chistoserdova et al., 2009; Chistoserdova, 2011). Thus, the MAGs of these ^13^C-labeled microorganisms indicated their genetic potential for XoxF-based methylotrophic metabolism.

**TABLE 2:** ^13^C-labeled proteins and microorganisms from ^13^C-methanol proteomic SIP.

**Labeled protein (Protein ID)**	**Day-3 (^13^C Atom%)**			**Day-8 (^13^C Atom%)**			**MAG ID**	**Completeness**	**Contamination**	**Taxonomy**	**Genome size (Mbp)**	**# of Genes**
	**Rep 1**	**Rep 2**	**Rep 3**	**Rep 1**	**Rep 2**	**Rep 3**						
Threonine-tRNA ligase (scaff_0000000132_40)	97	98	98	98	97	98	MAG47	43%	4%	Actinomycetales	3.4	3359
XoxF-type methanol dehydrogenase (scaff_0000127167_4)	55	55	59	72	68	59	MAG48	37%	0%	Rhizobiales	2.3	2418
XoxF-type methanol dehydrogenase (scaff_0000032941_13)	95	98	94	–	–	96	MAG49	17%	2%	Alphaproteobacteria	1.4	1477
SOS ribosomal protein L5 (scaff_0000001175_15)	93	–	93	–	–	–	MAG50	48%	5%	*Proteobacteria*	3.1	2996
XoxF-type methanol dehydrogenase (scaff_0000138535_1)	–	–	–	96	95	95	MAG51	8%	0%	*Proteobacteria*	0.3	300
Transaldolase (scaff_0000073426_7)	–	–	–	95	–	94	MAG52	23%	3%	Unclassified	1.4	1374
Protein with unknown function (scaff_0000002428_1)	–	–	–	84	–	84	MAG53	66%	4%	Rhizobiales	2.3	2354
Malate dehydrogenase (scaff_0000012663_3)	–	–	–	–	58	67	MAG54	31%	3%	Bradyrhizobiaceae	1.8	1781

## Discussion

In this study, proteomic SIP was combined with targeted metagenomic binning in two ^13^C SIP experiments on complex soil communities. Proteomic SIP was first used to identify ^13^C-labeled proteins with a low FDR. Then, targeted binning based on the abundance series of scaffolds across 14 samples generated MAGs for the microorganisms that produced the identified ^13^C-labeled proteins. Finally, the genetic potential and expressed functions of these microorganisms were inferred from their MAGs and identified proteins. In comparison with other SIP approaches, proteomic SIP was able to identify specific ^13^C-labeled microorganisms in a community and simultaneously reveal their partial genomes and proteomes.

This approach revealed four microorganisms that synthesized ^13^C-labeled proteins in the rhizosphere of plants growing under a ^13^CO_2_ atmosphere. The ^13^C-labeled microbial proteins indicated the transfer of photosynthetically fixed ^13^C from the plant hosts to these rhizosphere microorganisms. The ^13^C-labeled microorganisms expressed proteins for transport of carbohydrates and amino acids, which are known components of plant root exudates (Zhalnina et al., 2018). These compounds may be the ^13^C intermediates between the plant hosts and these ^13^C-labeled microorganisms. Besides the metabolic coupling, these ^13^C-labeled microorganisms had the genetic potential for extensive signaling interaction with the plant hosts by modulating plant hormones. In particular, the MAG44 encoded the genes involved in synthesizing cytokinin and auxin and suppressing ethylene via ACCD. This suggested that the ^13^C-labeled microorganism for MAG 44 can potentially modulate all these three plant hormones together to fine-tune the plant metabolic activities. Overall, our results supported many metabolic activities of rhizosphere microorganisms described in previous studies (Tien et al., 1979; Costacurta and Vanderleyden, 1995; Starr et al., 2018).

XoxF-type methanol dehydrogenase was found to be one of the most abundant proteins expressed by microbial communities in sea water (Sowell et al., 2011), grassland soil (Butterfield et al., 2016; Diamond et al., 2018), and phyllosphere of soybean, clover, and *Arabidopsis thaliana* (Delmotte et al., 2009), and in the rhizosphere soils of this study. The methanol oxidation activity of XoxF from pure cultures has been confirmed in laboratory (Schmidt et al., 2010). In rhizosphere soil, methanol may be released from plant roots as a by-product of pectin demethylation and lignin degradation (Galbally and Kirstine, 2002). In this study, proteomic SIP and metagenomics was used to establish a direct connection between the methanol addition and the biosynthesis of new XoxF proteins in methanol-utilizing microorganisms. The MAGs of these ^13^C-labeled methanol-utilizing microorganisms suggested that they can assimilate ^13^C-methanol via methanol oxidation, formaldehyde oxidation, formate oxidation, and the serine cycle. Proteomic SIP provided much stronger support for the methanol oxidation activity of XoxF than a potential comparative metaproteomics measurement that can only associate the substrate addition with protein abundance changes with or without new protein synthesis in organisms that may or may not directly utilize the added substrate. The ^13^C-methanol SIP experiment showed that proteomic SIP and metagenomics can link the consumption of a certain substrate with new biosynthesis of specific enzymes in organisms that have assimilated the substrate.

## Materials and Methods

### Plant Growth and ^13^CO_2_ Labeling

Twelve 0.25-L pots were filled with 330 g of a moistened loamy sand soil collected from a biological *Lolium perenne* grass field (51°59′28.0″N 5°39′43.1″E; upper 0–10 cm). The soil was sieved (5 mm) and subsequently adjusted to about 60% of field capacity (16%, w/w) by addition of deionized water. At day 0, seeds of *Zea mays* L. cv ‘Yukon Chief’ and *Triticum aestivum* L. cv ‘Baldus’ were germinated on wet tissue paper in petri dishes and transferred to the pots at Day 6 with two seedlings per pot. Ca. 30 seeds of *Arabidopsis thaliana* (L.) Heynh. cv ‘Columbia-O’ were directly sown in the pots. The plants were cultured in IsoLife’s (Wageningen, The Netherlands) stable isotope labeling facility under the following conditions: temperatures: 18/14°C (day/night), relative humidity: 75% (continuously), irradiance: 600 μmol⋅m^–2^ ⋅s^–1^ (PAR) with a day/night rhythm of 12/12 h, and CO_2_ level: 400 ppm. Nutrients were given as a half-strength Hoagland’s solution with corresponding N content of 10, 20, and 30 mg N per pot for *A. thaliana, T. aestivum, and Z. mays*, respectively. This fertilizer solution was given twice during the experiment, on Day 20, and on Day 33. From Day 30 till Day 38, the plants were labeled with 99 ^13^C atom% ^13^CO_2_. During the ^13^C-labeling period, the enrichment level decreased to ∼90 ^13^C atom% due to ^12^CO_2_ respiration from the plants. Three and eight days after the start of the ^13^C-labeling, half of the pots (6) were harvested. All shoots were cut at soil level. These shoots and the undisturbed soils were directly frozen and stored at −80°C prior to further processing.

### Rhizosphere Soil Harvesting

Rhizosphere samples were harvested from roots as previously described (Lundberg et al., 2012; Lebeis et al., 2015). Briefly, roots were removed from each pot and separated from loose soil. The roots with rhizosphere soil were transferred to a sterile 50-mL Falcon tube with 25 mL of sterile harvesting buffer (6.33 g of NaH_2_PO_4_.H_2_0, 16.5 g of Na_2_HPO_4_.H_2_0, and 200 μL Silwet L-77 in 1 L of water). The Falcon tubes were then vortexed for 15 s to separate the rhizosphere soil from the roots. Large roots were removed from the Falcon tubes using sterile tweezers. The turbid solutions were filtered through a sterile 100 μm nylon mesh cell strainer (BD Biosciences) into a new sterile 50-mL Falcon tube to remove sand and fine roots. The resulting turbid filtrates were centrifuged to generate pellets of rhizosphere soil. The initial soil samples collected from the sieved soil were processed in the same way as the rhizosphere soil samples as described above.

### ^13^C-Methanol Stable Isotope Probing

Fresh soil was collected identically from the same site as the soil used above in the plant growth experiment. Six microcosms were set up with 4 g of soil in glass jars. The ^13^C-methanol labeling solution was 5% v/v of ^13^C-methanol (99 atom% ^13^C, Sigma-Aldrich) in a half-strength Hoagland’s solution. Every day, 20 μL of the ^13^C-methanol labeling solution was added into each microcosm’s soil, which was then homogenized and incubated at the room temperature. The amount of ^13^C-methanol added to the soil communities in this study was substantially lower than a previous study of ^13^C-methanol SIP of soil communities (Radajewski et al., 2000). A triplicate of the microcosms was harvested after 3 days of ^13^C-methanol addition and incubation. Another triplicate was harvested after 8 days.

### DNA Extraction and Metagenomic Sequencing

Total DNAs in the collected soil samples were extracted using the PowerSoil DNA Isolation kit (Mo Bio Laboratories) in technical triplicates, which were pooled and purified using PowerClean Pro DNA Clean-Up kit (Mo Bio Laboratories). DNA concentrations were quantified with the Qubit fluorometric assay (Invitrogen) and 260/280 and 260/230 ratios were quantified using NanoDrop Spectrophotometer (Thermo Scientific). After TruSeq PCR-free library preparation, the 14 DNA samples (12 from the rhizosphere and 2 from the initial soil) were multiplexed together and sequenced by two 2 × 150-bp sequencing lanes on Illumina HiSeq 3000 at the Center for Genome Research and Biocomputing of Oregon State University.

### Metagenomic Assembly, Gene Prediction, and Functional Annotation

Metagenomic reads were pre-processed using BBTools^1^ for adapter sequence removal, read trimming and filtering, and sequencing error correction. The pre-processed reads from 14 samples were co-assembled to a composite metagenome with Omega 3.0 (Haider et al., 2014). Genes and proteins were predicted from scaffolds >1 kbp using the Prodigal (Hyatt et al., 2010). Protein functions were annotated using the UniFam protein family database (Chai et al., 2014).

### Untargeted Binning

Untargeted binning of scaffolds >5 kbp were performed with anvi’o v3 following the workflow described previously (Eren et al., 2015; Delmont et al., 2018). We used the anvi-cluster-with-concoct program which implemented the binning algorithm CONCOCT (Alneberg et al., 2014) to automatically cluster the scaffolds to 10 clusters. Then, we used the anvi-refine program to manually bin each CONCOCT cluster to generate MAGs with more than 70% completeness and less than 10% contamination estimated by anvi’o. We further estimated the quality of each MAG by checkM (Parks et al., 2015) and required each MAG to have more than 70% completeness and less than 10% contamination estimated by lineage-specific marker genes in checkM. The completeness and contamination of MAG43 was estimated by anvi’o because the MAG43 does not contain lineage-specific maker genes.

### Targeted Binning

Targeted binning followed the same procedures as the untargeted binning, except that the anvi-refine program was executed with the –additional-layers parameter to highlight the scaffolds which contain genes encoding ^13^C-labeled proteins in each CONCOCT cluster. We carried out manual binning around these highlighted scaffolds and extracted MAGs that had less than 5% contamination estimated by checkM and contained at least two ^13^C-labeled proteins identified in the rhizosphere or one ^13^C-labeled protein identified from at least two replicates in a SIP incubation triplicate in the ^13^C-methanol SIP experiment.

### Protein Extraction

Initial soils, rhizosphere soils, and methanol SIP microcosm soils were prepared for protein extraction in the same manner. One gram of soil from each sample was ground to fine powder in liquid nitrogen using a mortar and pestle. The ground soil sample was transferred to a 2-mL Eppendorf tube. One mL of the Solution SP1 of the NoviPure Soil Protein Extraction Kit (Mo Bio Laboratories) was added to the 2-mL tube which was then vortexed to completely mix the soil with the buffer, incubated at 4°C for 10 min, sonicated (20% amplitude, 10 s pulse with 10 s rest, 2 min total pulse time). After the sonication, 0.3 mL of the Solution SP2 was added into the tube which was then vortexed, incubated at 4°C for 30 min, and sonicated again using the same setting as described above. The tube was centrifuged at 10,000 × *g* for 5 min and the supernatant containing the crude protein extract was transferred into a new 2-mL Eppendorf tube. The crude protein extract in the supernatant was precipitated by trichloroacetic acid (Sigma-Aldrich) overnight at 4°C, pelleted by centrifugation, and washed with ice-cold acetone. The pelleted protein was re-suspended in 200 μL of 6 M guanidine (Sigma-Aldrich). In order to extract sufficient protein for triplicate metaproteomic measurements of each sample, duplicate protein extractions were conducted for each sample. The extracted proteins from the duplicate extractions of the same sample were pooled and quantified by BCA assay (Pierce Biotechnology). After the BCA assay, dithiothreitol (Sigma-Aldrich) was added to the tube for reducing the disulfide bonds of proteins. Fifty μg of proteins from each soil sample were further processed using the filter-aided sample preparation as described previously (Wiśniewski et al., 2009; Li et al., 2014). Proteins were first digested with trypsin (Promega) using an enzyme:substrate ratio of 1:100 (w:w) for overnight at room temperature, followed by an additional digestion with trypsin using the same enzyme:substrate ratio for 4 h. The peptide samples were stored at −80°C.

### Liquid Chromatography-Mass Spectrometry (LC-MS)

Metaproteomic measurements were carried out by using the 11-step multidimensional protein identification technology (MudPIT) (Washburn et al., 2001) on an LTQ-Orbitrap Elite mass spectrometer (Thermo Scientific), as described previously (Li et al., 2014). Ten microgram of proteins were loaded onto a MudPIT run. The MudPIT runs were configured with 11 salt pulses at 5, 7, 10, 12, 15, 17, 20, 25, 35, 50, and 100% of Solvent D which contains 500 mM ammonium acetate dissolved in the Solvent A (95% water, 5% acetonitrile and 0.1% formic acid). Each salt pulse was followed by a 110-min gradient from 100% of the Solvent A to 60% of the Solvent B (30% water, 70% acetonitrile, and 0.1% formic acid). The LC eluent was directly nanosprayed (Proxeon) into the LTQ Orbitrap Elite mass spectrometer. MS scans were acquired in Orbitrap with a resolution of 30,000. The top 8 most abundant ions were selected for collision-induced dissociation and fragment ions were measured in Orbitrap with a resolution of 15,000. Each soil sample had technical triplicate LC-MS measurements.

### Peptide and Protein Identification by Database Searching

The regular database searching for identification of unlabeled proteins was conducted using Sipros Ensemble (Guo et al., 2017). The soil microbial protein database comprised of >1.8 million proteins predicted from scaffolds >5 kbp in the composite metagenome assembly. The initial soil samples were searched against the soil microbial protein database. An *A. thaliana proteome* database downloaded from https://phytozome.jgi.doe.gov/pz/portal.html#!bulk?org=Org_Athaliana (Araport11) was added to the soil microbial protein database to search the *A. thaliana* rhizosphere soil samples. A *Z. mays* proteome database from https://phytozome.jgi.doe.gov/pz/portal.html#!bulk?org=Org_Zmays (Ensembl-18) was added to the soil microbial protein database to search the *Z. mays* rhizosphere soil samples. A *T. aestivum* proteome database from https://phytozome.jgi.doe.gov/pz/portal.html#!bulk?org=Org_Taestivum_er (v2.2) was added to the soil microbial protein database to search the *T. aestivum* rhizosphere soil samples. Plant proteins identified in the rhizosphere samples were shown in Supplementary Table 5. The methanol incubation soil samples were searched again the soil microbial database. All these protein databases were constructed with concatenated forward and reverse proteins for FDR estimation. The searches used the following parameters: parent mass offsets of −1, 0, 1, 2, 3 Da; mass tolerance of 0.03 and 0.01 Da for parent ions and fragment ions, respectively; up to three missed cleavages; dynamic modification of methionine residue by oxidation; and full enzyme specificity required. The database searching results were filtered to achieve 1% FDR at the peptide level. Proteins were inferred from the identified peptides using the parsimony rule (Nesvizhskii and Aebersold, 2005). Indistinguishable proteins were combined as a protein group. A minimum of one peptide unique to each inferred proteins or protein groups was required. The FDR at the protein level was estimated to be less than 2% for all metaproteome samples. The abundance of identified proteins was quantified based on normalized peak areas using ProRata (Pan et al., 2006).

The SIP database searching for identification of ^13^C-labeled proteins was conducted using Sipros with the weighted dot-production scoring function as described in previous studies (Pan et al., 2011; Justice et al., 2014; Bryson et al., 2016, 2017; Marlow et al., 2016). The SIP database for the rhizosphere samples of each plant species included all the proteins identified from these samples in the regular searches. Similarly, the SIP database for the methanol incubation samples included all the proteins identified in these samples in the regular searches. SIP searches were also conducted for the initial soil samples as a negative control using a protein database containing all the unlabeled proteins identified in the initial soil samples. All SIP searches used the following parameters: parent mass offsets of −4, −3, −2, −1, 0, 1, 2, 3, 4 Da; 0.03 Da of mass tolerances for parent ions; 0.01 Da of mass tolerances for fragment ions; up to three missed cleavages; full enzyme specificity required. SIP searching results were filtered to achieve 1% FDR at the peptide level using a target-decoy approach (Elias and Gygi, 2007). Proteins were inferred from the peptide identification results by requiring a minimum of one unique peptide per protein or protein group. Each ^13^C-labeled protein was required to have a minimum of >10% ^13^C enrichment and it also must have a minimum of two peptides identified in at least one of the four rhizosphere samples of the same plant species with which it was associated. ^13^C-labeled peptides were clustered into protein isotopologues until the average atom% difference between clusters was >10%.

### Taxonomy Assignment

The taxonomy of MAGs were initially inferred using three different tools, namely, checkM (Parks et al., 2015), AMPHORA (Wu and Eisen, 2008), and best USEARCH (Edgar, 2010) hits of predicted proteins on MAG scaffolds to the Uniprot, Uniref90, and KEGG databases. The AMPHORA-based taxonomy assignment of these MAGs was conducted on the AmphoraNet webserver (Kerepesi et al., 2014) and each MAG was assigned with a taxon if at least 75% of the identified marker genes in that MAG generated a concordant taxonomy or with a putative taxon if more than 50% and less than 75% of the identified marker genes generated a concordant taxonomy (Muller et al., 2014). If the taxonomy assignments of a MAG from at least two of the three approaches agreed at the phylum level, the lowest common ancestor of these taxonomy assignments were selected as the taxon of that MAG.

### Phylogenomic Analysis

The root-associated isolate genomes were downloaded from the Supplementary Material of Levy et al. (2018). We used Phylosift (Darling et al., 2014) to extract marker genes from all the MAGs and the root-associated isolated genomes. A phylogenomic tree of these genomes was constructed based on the concatenated protein sequence alignment of marker genes using the program anvi-gen-phylogenomic-tree in anvi’o and the FastTree algorithm (Price et al., 2010). The tree was uploaded into the Interactive Tree Of Life server for visualization.

### Phylogenetic Analysis of XoxF

The protein sequences of 68 XoxF identified by metaproteomics in this study were aligned to 143 reference XoxF sequences obtained from a previously study (Taubert et al., 2015) using Clustal Omega at https://www.ebi.ac.uk/Tools/msa/clustalo/. A newick-formatted tree was generated after the sequence alignment. The tree was uploaded into the Interactive Tree Of Life server for visualization.

## Data Availability Statement

Metagenomic sequencing reads associated with this study have been deposited in BioProject under accession PRJNA488251. Mass spectrometry raw files for this study have been deposited in ProteomeXchange under accessions PXD011737, PXD011738, PXD011739, PXD011891, and PXD011892. MAGs recovered in this study can be downloaded from https://figshare.com/s/2a812c513ab14e6c8161.

## Author Contributions

CP, ZL, and GH designed the study. ZL, QY, MM, RH, and SL performed the experiments. ZL, QY, XG, WH, and CP implemented data analysis algorithms. ZL, QY, A-CC, and JB analyzed metagenomics binning results. ZL and CP analyzed the results and prepared the manuscript. All authors discussed the results and edited the manuscript.

## Disclaimer

This manuscript has been authored in part by UT-Battelle, LLC, under contract DE-AC05-00OR22725 with the U.S. Department of Energy (DOE). The U.S. Government retains and the publisher, by accepting the article for publication, acknowledges that the U.S. Government retains a non-exclusive, paid-up, irrevocable, worldwide license to publish or reproduce the published form of this manuscript, or allow others to do so, for U.S. Government purposes. DOE will provide public access to these results of federally sponsored research in accordance with the DOE Public Access Plan (http://energy.gov/downloads/doe-public-access-plan). The opinions and assertions contained herein are those of the authors and are not to be construed as those of the U.S. Government.

## Conflict of Interest

The authors declare that the research was conducted in the absence of any commercial or financial relationships that could be construed as a potential conflict of interest.

## Footnotes

1 http://jgi.doe.gov/data-and-tools/bbtools/
