# Supplementary material for: Genome-Resolved Proteomic Stable Isotope Probing of Soil Microbial Communities Using 13CO2 and 13C-Methanol
Source: Front Microbiol. 2019 Dec 6;10:2706. doi: 10.3389/fmicb.2019.02706 (PMC6908837; doi:10.3389/fmicb.2019.02706)
Supplement: Supplementary file 6 [file Presentation_1.pdf]

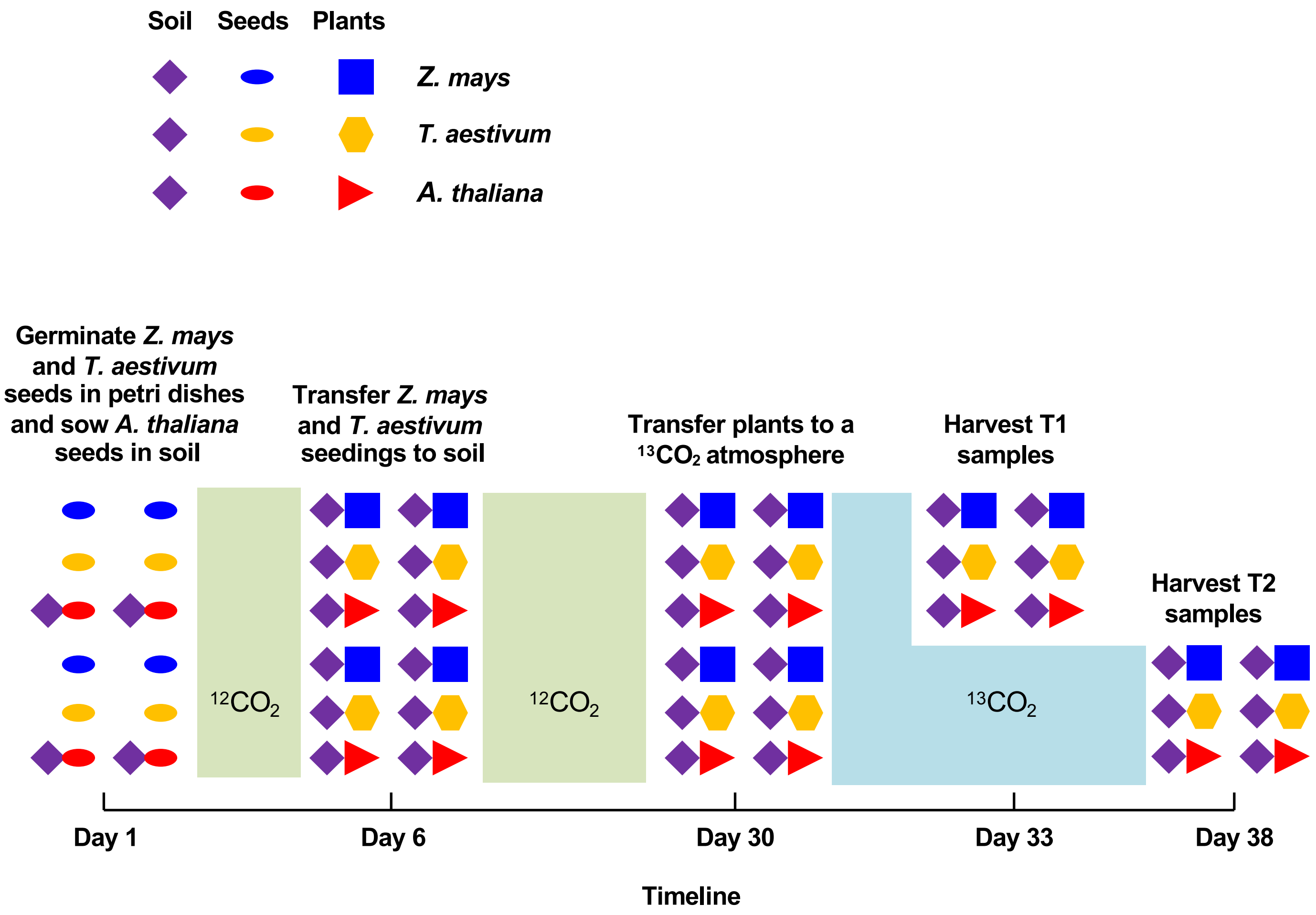

Supplementary Figure 1: Experimental design of the $^{13}CO_2$ rhizosphere proteomic SIP experiment. *Z. mays*, *T. aestivum*, and *A. thaliana* plants were grown in a normal atmosphere from Day 1 to Day 29. These plants were moved to a $^{13}CO_2$ atmosphere on Day 30. Two pots per plant species were harvested on Day 33 as timepoint-1 samples (T1) and on Day 38 as timepoint-2 samples (T2).

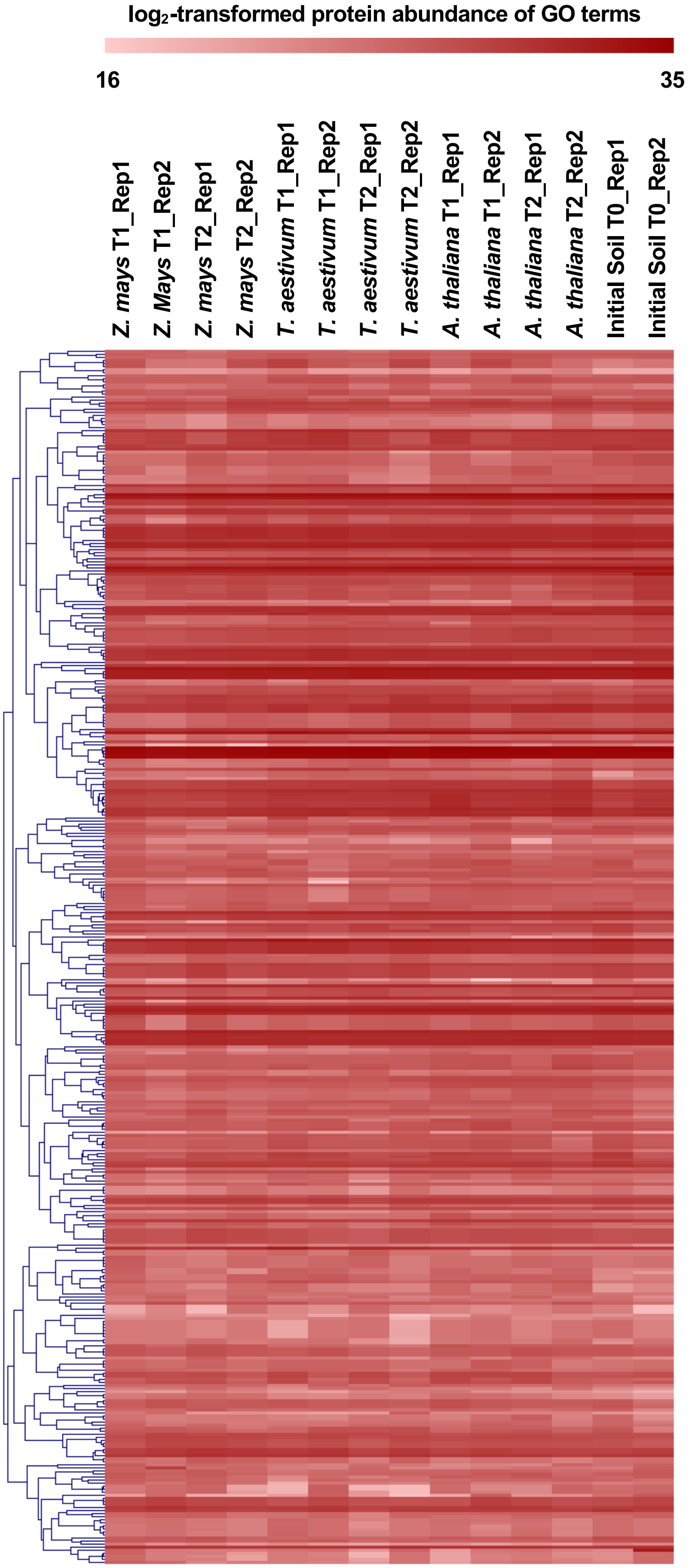

Supplementary Figure 2: Heatmap of $\log_2$-transformed protein abundances of GO terms of rhizosphere and initial soil samples. GO terms identified across all twelve rhizosphere and two initial soil communities were clustered by their unlabeled protein abundances measured by metaproteomics.

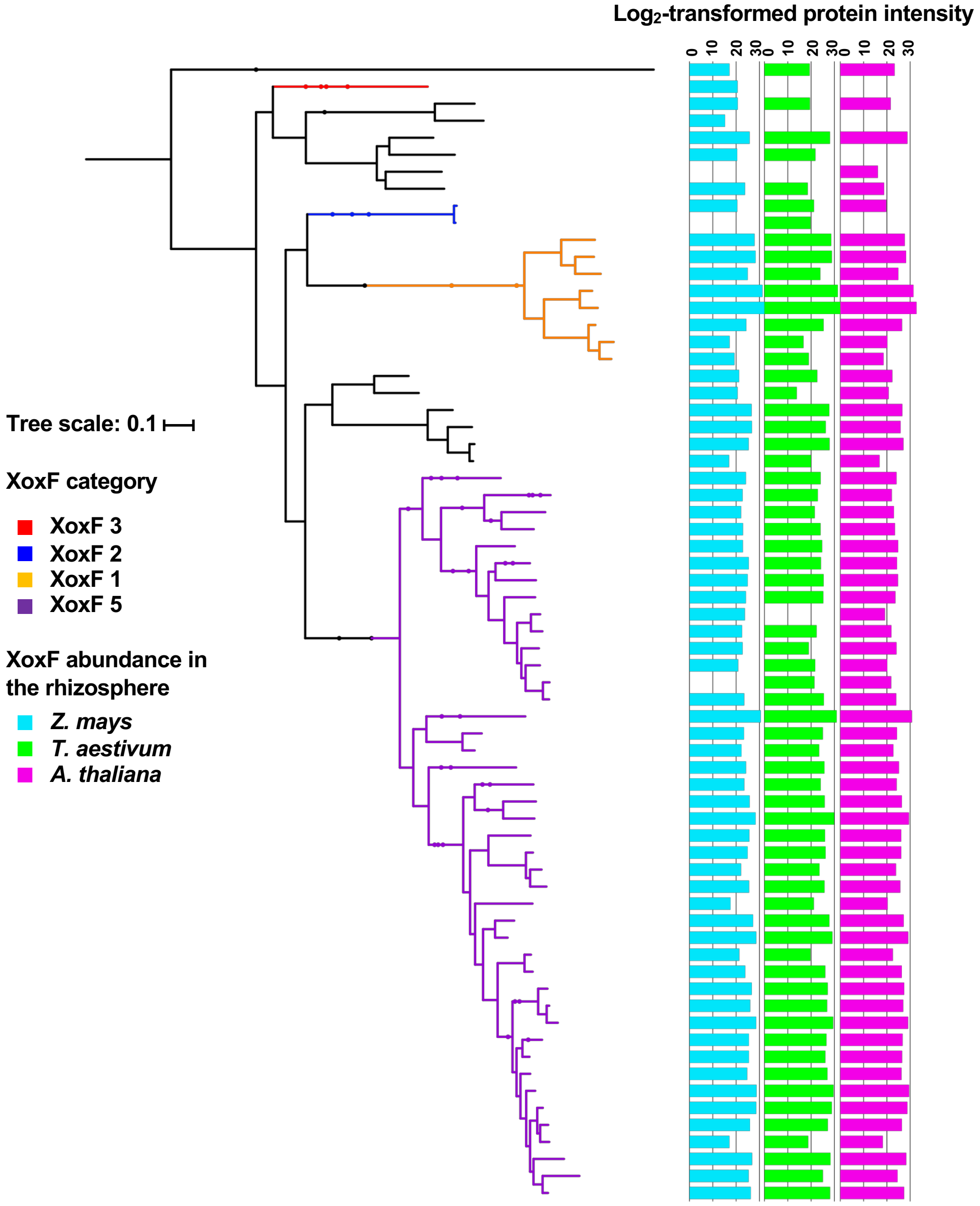

Supplementary Figure 3A: Phylogenetic tree of 68 XoxF proteins identified by metaproteomics in this study in relation to 146 reference XoxF sequences. Only branches of the 68 XoxF proteins are shown in the figure and branches of the 146 reference sequences are deleted. See the supplementary figure 3 for the full tree. Each branch is color-coded according to the XoxF category established in Taubert et al. The bar graphs represent the log_2-transformed XoxF protein abundance in the rhizosphere of each plant species.

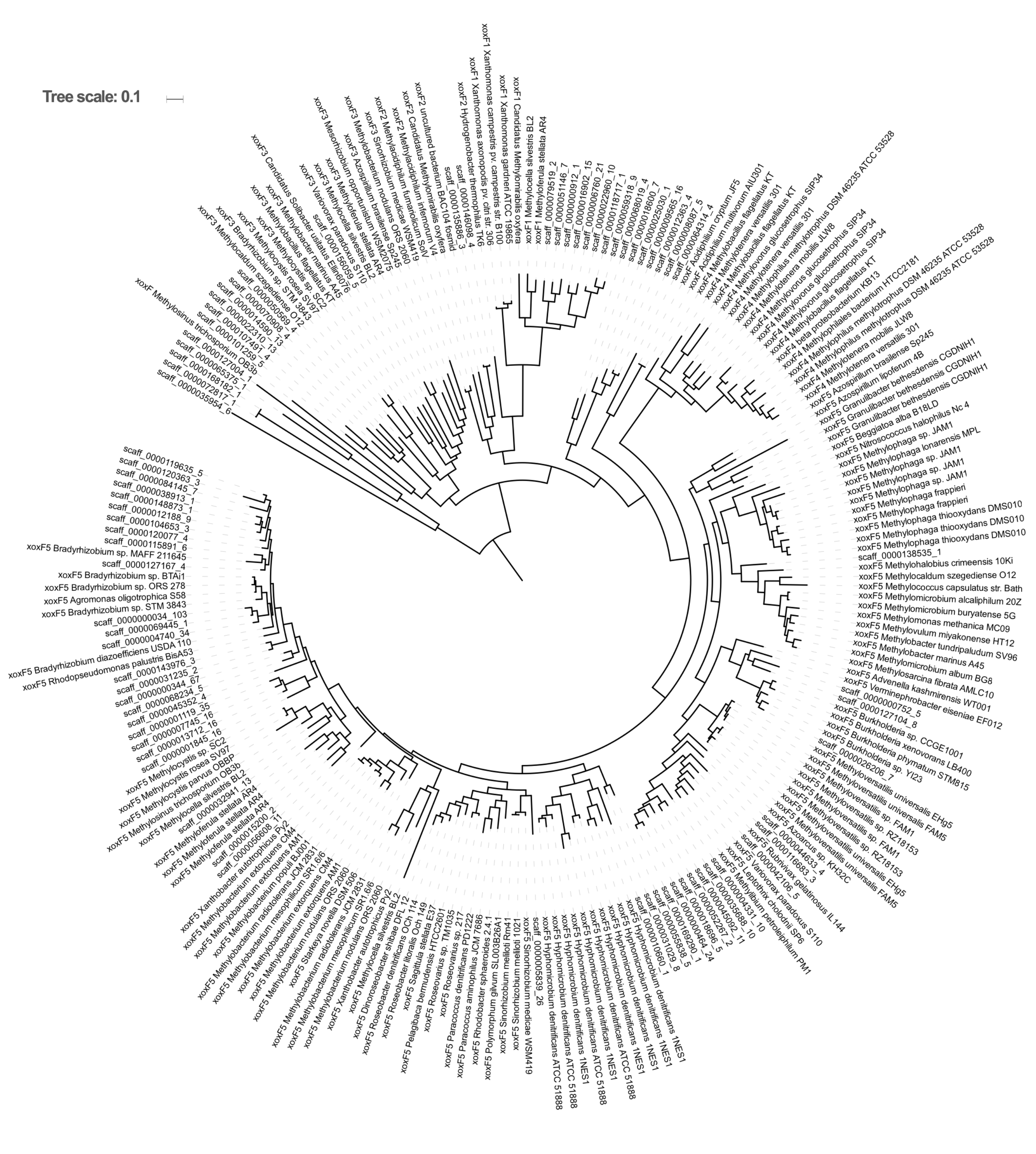

Supplementary Figure 3B: Full phylogenetic tree of XoxF proteins identified by metaproteomics in this study in relation to 146 reference XoxF sequences from Taubert et al. Branches of XoxF proteins identified from this study are labeled with Protein ID starting with “scaff”. The rest of the branches belong to reference sequences.

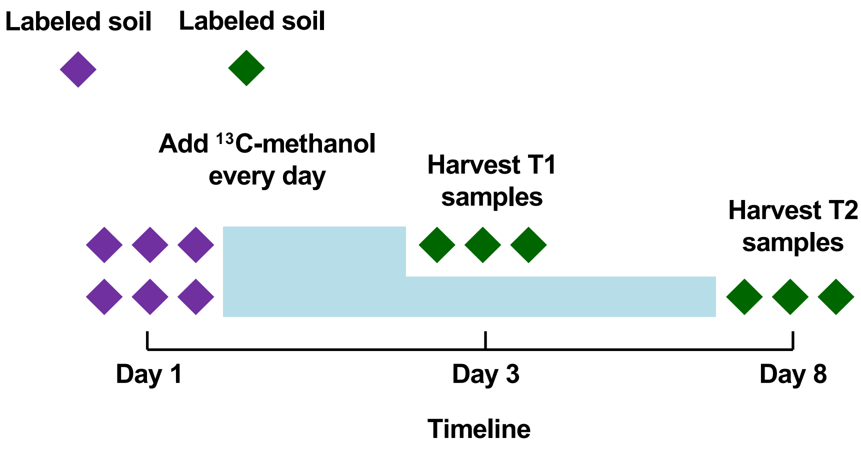

Supplementary Figure 4: Experiment design of the ^{13}C-methanol proteomic SIP experiment. ^{13}C-methanol was added into six replicates of soil microcosms every day during the labeling. Triplicate microcosms were harvested after 3 days (T1) of incubation and after 8 days (T2) of incubation.
